# Enhancer of rudimentary homolog regulates DNA damage response in hepatocellular carcinoma

**DOI:** 10.1038/srep09357

**Published:** 2015-04-09

**Authors:** Meng-Tzu Weng, Tzu-Hsun Tung, Jih-Hsiang Lee, Shu-Chen Wei, Hang-Li Lin, Yu-Jung Huang, Jau-Min Wong, Ji Luo, Jin-Chuan Sheu

**Affiliations:** 1 Graduate Institute of Clinical Medicine, National Taiwan University, Taipei 100, Taiwan; 2 Far-Eastern Memorial Hospital, New Taipei 220, Taiwan; 3 Liver Disease Prevention and Treatment Research Foundation, Taipei 100, Taiwan; 4 Clinical Trial Center, Taipei 100, Taiwan; 5 Department of Internal Medicine, National Taiwan University Hospital and College of Medicine, Taipei 100, Taiwan; 6 Cancer Systems Biology Section, Laboratory of Cancer Biology and Genetics, National Cancer Institute, NIH Bethesda, MD 20892, USA

## Abstract

We previously demonstrated that the *enhancer of rudimentary homolog (ERH)* gene is required for the expression of multiple cell cycle and DNA damage response (DDR) genes. The present study investigated the role of ERH and its target DNA damage repair genes in hepatocellular carcinoma cells. We observed positive correlation between ERH and ataxia telangiectasia and Rad3 related (ATR) expression in liver tissues. Expression of ERH, ATR as well as checkpoint kinase 1 (CHK1) were higher in HCCs than in normal liver tissues. Knocking-down ERH augmented ultraviolet light induced DNA damage in HepG2 cells. ATR protein level is reduced upon ERH depletion as a result of defect in the splicing of ATR mRNA. Consequently, the ATR effector kinase Chk1 failed to be phosphorylated upon ultraviolet light or hydroxyurea treatment in ERH knocked-down HepG2 cells. Finally, we observed Chk1 inhibitor AZD7762 enhanced the effect of doxorubicin on inhibiting growth of HCC cells in vitro and in vivo. This study suggested that ERH regulates the splicing of the DNA damage response proteins ATR in HCC cells, and targeting DNA damage response by Chk1 inhibitor augments chemotherapy to treat HCC cells.

Hepatocellular carcinoma (HCC) is the most common primary liver cancer and is the third leading cause of cancer-related death worldwide^1^,^2^. Sorafenib is the only approved systemic therapy for advanced HCC, but the median survival of HCC patients is still less than one year^3^. No systemic chemotherapy has shown to improve survival of advanced HCC^2^,^4^, and doxorubicin, a topoisomerase inhibitor which damages DNA, is widely used to treat advanced HCC^4^.

Genetic alterations are common in HCCs^5^,^6^. The DNA damage response (DDR) pathway is essential for maintenance of genomic integrity during replication and in situations of genomic stress. Dysregulation of DDR is often involved in the carcinogenesis of HCC and may contribute to HCC's resistance to chemotherapies^7^. Gene expression analysis has demonstrated up-regulation of DNA repair genes involved in the activation of ataxia telangiectasia and Rad3 related (ATR) kinase in HCC cells^8^. Thus, the DNA repair pathway could be a potential target for cancer therapy against HCC^9^.

Enhancer of rudimentary homolog *(ERH)*, originally identified in *Drosophila*, is a highly conserved gene among metazoans^10^. We previously demonstrated that ERH is a novel splicing factor that regulates the mRNA splicing of the mitotic motor protein CENP-E, and knocking-down ERH in cancer cells resulted in chromosome congression defects during mitosis^11^. Analysis of changes in gene expression profile in colorectal cancer cells upon ERH depletion revealed the down-regulation of several additional cell cycle genes, including *ATR*^11^. ATR is a DNA damage checkpoint kinase that is activated by single stranded DNA breaks and by replication stress, and it is required for cell cycle arrest in response to DNA damage. ATR phosphorylates the checkpoint kinase 1 (CHK1), and other checkpoint proteins including RAD17 and the tumor suppressor protein BRCA1^12^,^13^. CGK733, a small molecule inhibitor targeting the ATR, significantly enhances paclitaxel-induced cytotoxicity in a HCC cell line^14^. However, little is known about the role of ERH in DNA damage response, and its role in HCCs.

In the current study, we evaluated the roles of ERH to DNA damage response in HCC cells and we studied whether ERH and its target DNA damage response genes could be a potential therapeutic target in HCC cells.

## Results

### ERH and ATR expression are elevated in HCC tumors

**Figure 1:**
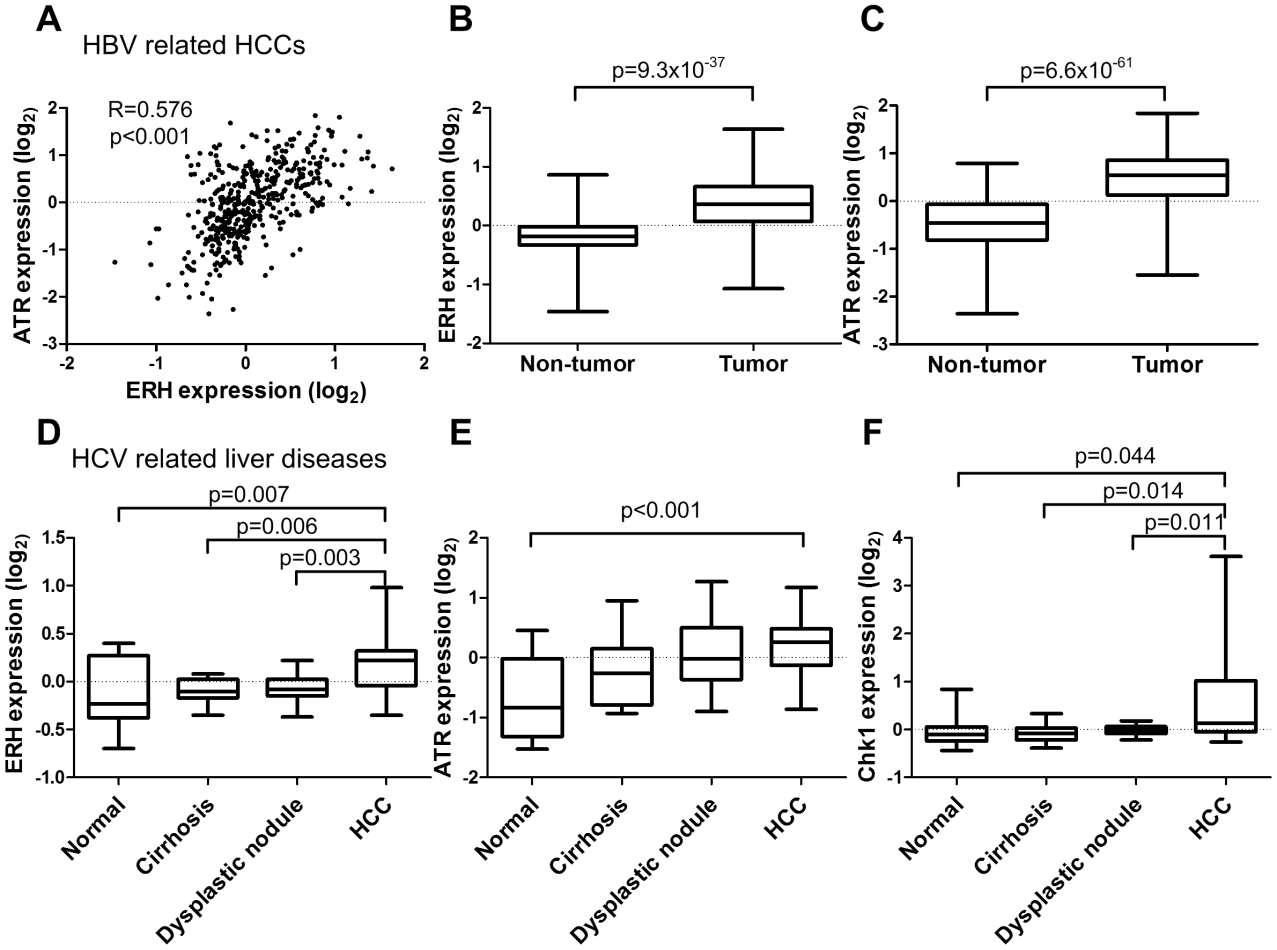
ERH expression in HBV and HCV related HCC and non-tumor liver tissue. (A) Relationship between mRNA expression of ERH and ATR in liver tissue (correlation coefficient = 0.576, p < 0.001 by Spearman's method). (B) ERH mRNA and (C) ATR mRNA expression in tumor part and non-tumor part in HBV related HCCs. (D) ERH mRNA, (E) ATR mRNA, and (F) CHK1 mRNA expression in HCC, normal liver, cirrhosis and dysplastic tissue of HCV carriers. The p-values were less than 0.001 by one-way ANOVA for the comparison of all 3 genes. The *p*-values demonstrated in the panels were the results of post-hoc analysis by Bonferroni method.

To investigate the role of ERH in regulating the DDR, we first examined the expression of ERH and ATR mRNA in HCC tumors using publicly accessible gene expression databases. The GSE14520 dataset^15^ contains gene expression profiles from hepatitis B (HBV) positive HCCs and matching normal liver tissues. In this dataset, expression of ERH mRNA was correlated with expression of ATR mRNA in 444 liver tissues containing HCC and non-tumor tissues (Figure 1A). ERH mRNA expression in tumor tissue was higher than in the matched normal tissue (p = 9.3 × 10^−37^) (Figure 1B). Similarly, ATR expression was also higher in tumor tissue compared to matched normal controls (p = 6.6 × 10^−61^) (Figure 1C).

We next analyzed the GSE6764 dataset^16^, which contains gene expression profiles of hepatitis C (HCV) positive HCCs and normal liver tissue controls. In this dataset we also observed a correlation between ERH and ATR mRNA expression (correlation coefficient = 0.285, p = 0.013). We further observed that ERH mRNA expression was significantly higher in HCCs (n = 35) than in normal tissues (n = 10), cirrhosis (n = 13) or dysplastic lesions (n = 17), indicating that the transition from premalignant lesions to small HCC is associated with an increase of ERH expression (Figure 1D). Similarly, in these cases, ATR mRNA expression was significant higher in HCCs than in normal liver tissues (p < 0.001 by one-way ANOVA and p < 0.001 for HCC vs. normal tissue with post-hoc analysis by Bonferroni way) (Figure 1E). Chk1 is a downstream effector kinase of ATR, we thus also investigated Chk1 expression in these samples. Similar to ATR, Chk1 mRNA expression is correlated with that of ERH (correlation coefficient = 0.504, p < 0.001), and Chk1 mRNA expression was higher in HCCs compared to normal tissues, cirrhosis tissue or dysplastic lesions (Figure 1F).

### ERH depletion augmented ultraviolet light (UV)-irradiation induced DNA damage in HCC cells

**Figure 2:**
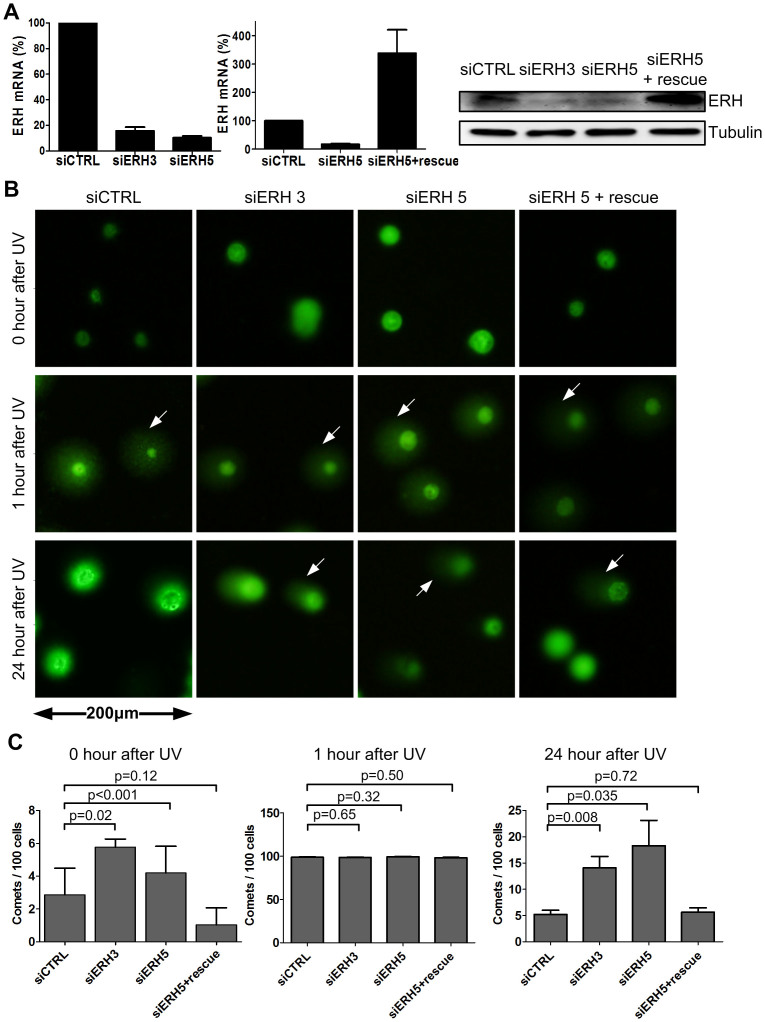
Knocking-down ERH impaired DNA-damage-repair ability. (A) Expression of ERH mRNA and protein in HepG2 cells upon ERH siRNA transfection and in ERH open reading frame stably expressing HepG2 cells upon siRNA ERH 5 transfection (siERH 5 rescue). (B) Representative images of comet assay in HepG2 cells upon ERH knocking-down and ERH rescue, followed by one episode of UV 200 J/m2 irradiation. Cells were collected at 0, 1 and 24 hours after irradiation. (White arrows indicated damaged DNA) (C) Quantification of comet assay in figure 2B.

We next assessed the effect of ERH depletion on the DDR in HCC cells. We knocked down ERH in HepG2 cells using two ERH siRNAs that we had previous described^11^: one targeting the coding region of ERH mRNA (siERH-3) and one targeting its 3'-UTR (siERH-5) (Figure 2A, left panel). And as we showed previously^11^, stable expression of an ERH cDNA was able to selectively rescue against siERH-5 knockdown but not siERH-3 knockdown (Figure 2A, middle and right panels).

To investigate how ERH might influence DNA damage repair in HCC cells, we induced DNA damage using UV irradiation in cells with or without ERH depletion and measured the extent and time course of DNA damage repair using the comet assay^17^. We quantified DNA damage response by measuring the fraction of cells with nuclear DNA comet tails which is indicative of DNA breaks, at 0, 1 and 24 hours after UV. Knocking-down ERH in the HepG2 cells induced a modest increase of comets cells (Figure 2C, left panel). We also observed an increase of G2/M fraction of cell cycle in ERH knocked-down HCC cells (Figure S1A). Upon UV irradiation for 1 hour, almost all cells presented as having nuclear comet tails. The number of comet tails in cells transfected with control siRNA decreased to baseline level at 24 hours post UV, indicative that these cells have repaired their DNA damage. In contrast, in ERH knocking-down cells we observed a significant numbers of cells with comet tail at this later time point, indicative of persistent DNA break. This phenotype can be fully rescued by ERH cDNA, thus ruling out siRNA off-target effect (Figure 2B–C). Together these findings show that loss of ERH attenuated UV-induced DNA damage repair in HCC cells.

### ERH regulates ATR mRNA splicing and expression

**Figure 3:**
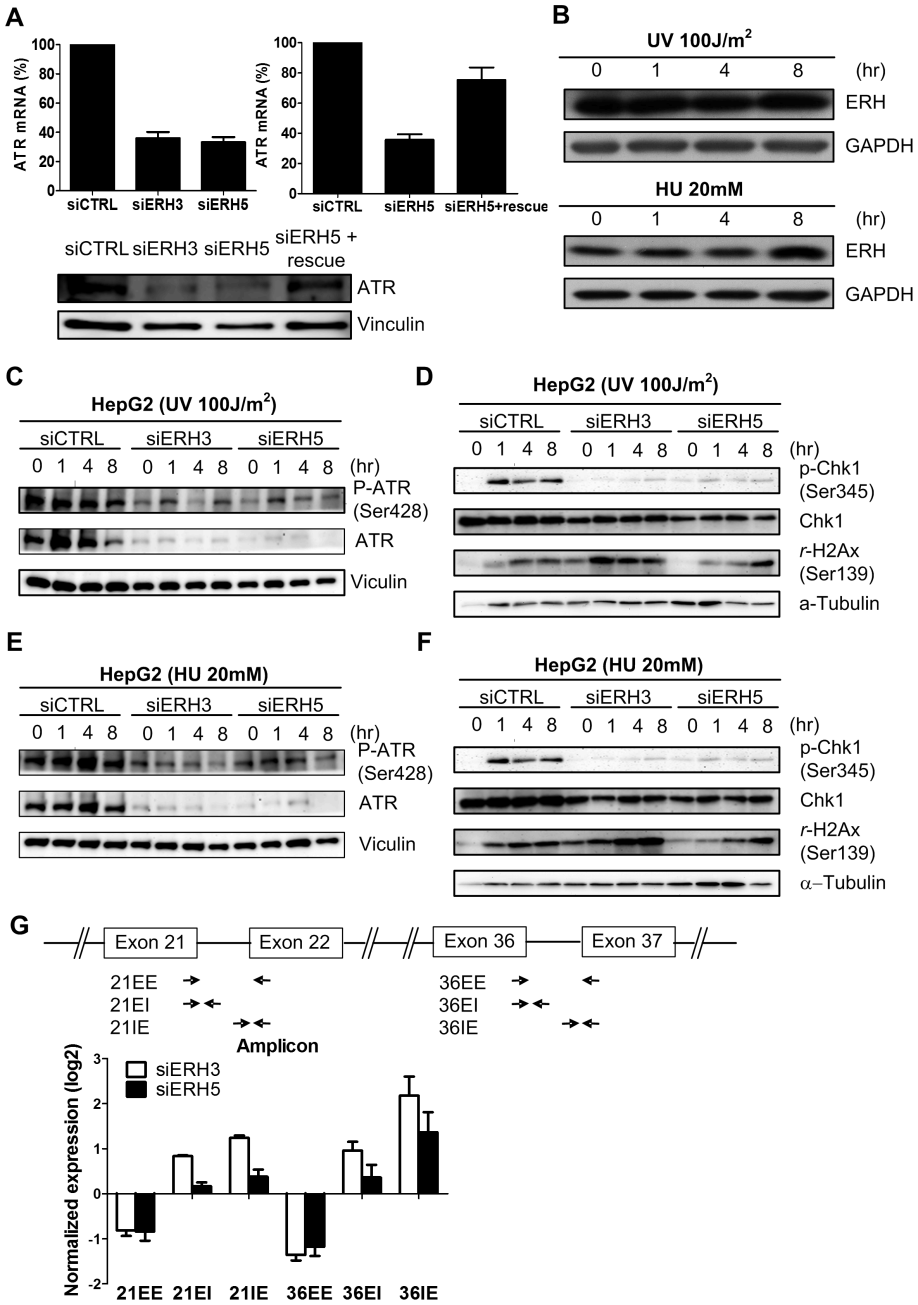
ERH modulated DNA damage response in HCC cells. (A) Expression of ERH mRNA and protein in HepG2 cells upon ERH siRNA transfection and in ERH open reading frame stably expressing HepG2 cells upon siRNA ERH 5 transfection (siERH 5 rescue). (B) ERH protein expression didn't change after UV 100 J/m2 irradiation or hydroxyurea 20 mM treatment. (C)Protein expression of total and phosphorylated ATR protein upon ERH knocking-down in HepG2 cells after UV 100 J/m2 irradiation of various durations. (D) Protein expression of total and phosphorylated Chk1 protein and gamma-H2AX expression upon ERH knocking-down after UV irradiation. (E) Protein expression of total and phosphorylated ATR protein upon ERH knocking-down in HepG2 cell upon hydroxyurea 20 mM treatment of various durations. (F) Protein expression of total and phosphorylated Chk1 protein and rH2Ax expression upon ERH knocking-down after hydroxyurea treatment. (G) ATR pre-mRNA expression at the exon–intron junctions detected by qPCR with exon-spanning and splice junction primers. The schematic indicates the location of PCR primers (EE, exon–exon PCR; EI, exon–intron PCR; IE, intron–exon PCR).

We previously demonstrated, using gene expression microarray, that ERH regulates ATR expression^11^. This phenomenon was also observed in both colon cancer and lung cancer cell lines (Figure S2A). We confirmed that ATR mRNA and protein expression was decreased upon transfection with ERH siRNAs in HCC cells. Once again this can be rescued by ERH cDNA, (Figure 3A), indicating that this is an on-target effect of ERH siRNAs. As ATR responds to replication stress and single-stranded DNA breaks^18^,^19^, we investigated whether knocking down ERH would affect ATR-mediated response to DNA damage in HCC cells treated with UV-radiation or with hydroxyurea (HU). First, we tested whether UV radiation or HU affect the ERH expression and found the protein level not change under treatment (Figure 3B). In cells transfected with control siRNA, UV and HU treatment led to a robust increase in phosphor-ATR in HepG2 cells, whereas ATR phosphorylation is largely abrogated in cells transfected with ERH siRNAs (Figure 3C and 3E), likely due to the loss of ATR protein in ERH depleted cells. Knocking-down ERH did not affect the expression of Chk1 (Figure S2B), but abrogated Chk1 activation as measured by its phosphorylation at the ATR-site S345 (Figure 3D and 3F). Comparable levels of γ-H2AX signals in HepG2 cells transfected with either control siRNA or ERH siRNAs indicated that this is not due to a difference in the extent of DNA damage (Figure 3D and 3F), but rather a failure of DNA damage to activate ATR signaling in these cells.

In our previous study, we demonstrated that ERH is a splicing factor that regulates CENP-E mRNA expression via controlling its splicing^11^. We thus tested whether ERH also regulated the splicing of ATR mRNA by measuring splicing efficiency across intron 21 and intron 36. We found that, at both introns, mature mRNA level was decreased whereas unspliced mRNA level was concomitantly increased in ERH depleted cells (Figure 3G). Thus ATR mRNA splicing is subject of regulation by ERH and the loss of mature ATR mRNA could account for the loss of ATR protein upon ERH depletion. As ERH and SNRPD3 are binding partners, we also knocked down SNRPD3 and found that led to ATR mRNA and protein expression partially loss (Figure S2C and S2D).

### CHK1 inhibitor AZD 7762 synergizes with doxorubicin to inhibit HCC cell proliferation

**Figure 4:**
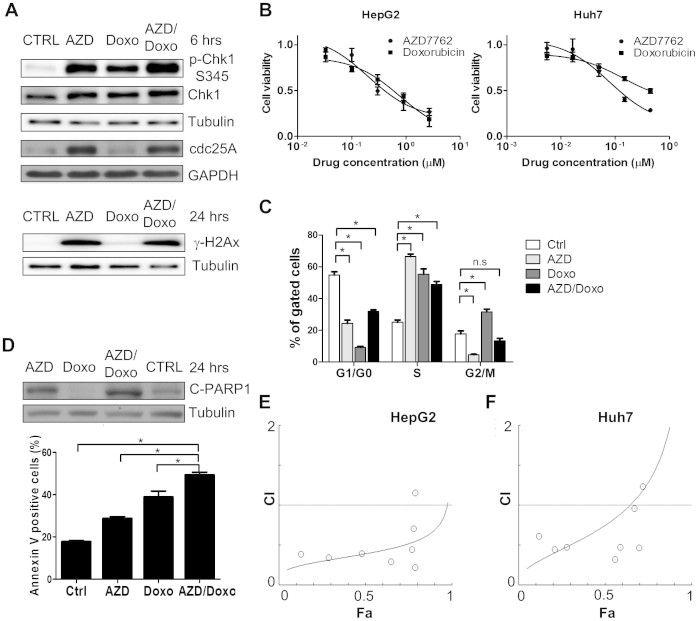
Effect of a combination of AZD7762 and doxorubicin in HCC cancer cell lines. (A) Protein expression of γ-H2AX, cdc25 A, total and phosphorylated Chk1 S345 protein in HepG2 cells upon 6 hours of AZD7762 (AZD) 500 nM, doxorubicin (Doxo) 500 nM or combination therapy. CTRL: media only. (B) Cell viability of HepG2 cells and Huh7 cells 72 hours after various concentrations of AZD7762 or doxorubicin treatment. (C) Cell cycle distributions in the Huh7 cells 72 hours after treatment of AZD7762 500 nM, doxorubicin 500 nM, or combination therapy. (D) Apoptosis of cell, as demonstrated by protein expression of cleaved PARP1 and percentage of annexin-V positive cells, in HepG2 cells upon AZD7762, doxorubicin or combination therapy. Cells were incubated for 48 hours for annexin-V experiment. (E) Combination index (CI) in HepG2 cells upon treatment with AZD7762 and doxorubicin. The CI was calculated using cell viability data presented in the supplementary figure S2A (F) CI in Huh7 cells upon treatment with AZD7762 and doxorubicin. The CI was calculated using cell viability data presented in the supplementary figure S2B. (n.s: non-specific, *: p < 0.05).

The elevated expression of ERH, ATR and CHK1 in HCCs suggests that in HCC cells combining DNA damage checkpoint inhibitors with DNA damage agents might lead to improve toxicity. We thus tested whether interfering with DNA damage response signaling could potentiate the cytotoxic effect of DNA damage agents. Knocking-down ERH by siRNAs was unable to potentiate HCC cells sensitivities to doxorubicin (Figure S3A). As ERH is undruggable, we next tested whether the Chk1 inhibitor AZD7762^20^ could synergize with the DNA damage agent doxorubicin in HCC cells. We observed stabilization of cdc25A in HepG2 cells upon AZD7762 treatment (Figure 4A) as previous reported^21^, indicating successful inhibition of Chk1 signaling by AZD7762. As expected, treatment with doxorubicin or AZD7762 activated the DDR and resulted in phosphorylation of Chk1 at serine 345, which is an ATR regulated site (Figure 4A). We also observed increased γ-H2AX in HepG2 cells upon AZD7762 and combination treatment for 24 hours, suggesting DNA damage in HepG2 cells (Figure 4A). The findings were consistent with the AZD7762 treatment effect in pancreatic cancer cells^22^. Both AZD7762 and doxorubin inhibited proliferation of HepG2 and Huh7 cells. The IC_50_ to AZD7762 and Doxorubicin were 0.401 μM and 0.583 μM, respectively, for HepG2 cells, and 0.118 μM and 0.427 μM, respectively, for Huh7 cells (Figure 4B). In Huh7 cells, AZD7762 induced S phase arrest and doxorubicin induced G2/M arrest. Combination of AZD7762 and doxorubicin predominantly leads to an S phase arrest phenotype that more closely resembles AZD7762 (Figure 4C). Higher expression of cleaved PARP1 and more apoptosis cells were observed in combination treatment group compared to other groups (Figure 4D).

We next tested whether AZD7762 and doxorubicin could work in synergy in HCC cells. The combination index (CI) of AZD7762 and doxorubicin were less than 1.0 mostly in HepG2 cells (Figure 4E, Figure S3B) and Huh7 cells (Figure 4F, Figure S3C), suggesting a synergistic effect in vitro.

### AZD 7762 enhanced the effect of doxorubicin on HCC xenografts

**Figure 5:**
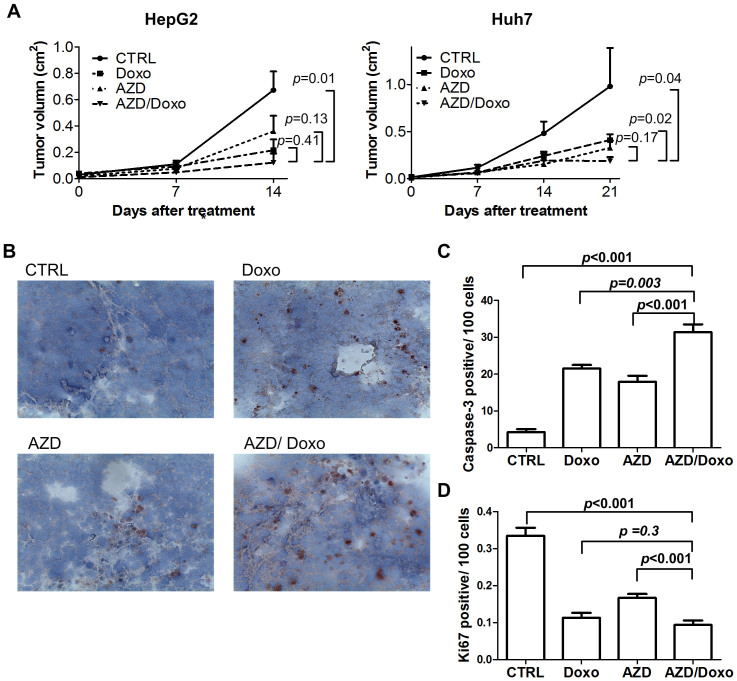
Effect of doxorubicin and/or AZD7762 on HCC xenografts. (A) Tumor volumes of HepG2 and Huh7 xenografts upon doxorubicin (Doxo) and/or AZD7762 (AZD) treatment. N = 5 for each group. Ctrl: vesicle. P < 0.05 for tumors on day 14 (HepG2) or day 21 (Huh7) of treatment by one-way ANOVA test, vertical bar: standard error. (B) Representative images of cleaved caspase-3 (dark brown) in HepG2 xenografts. (C) Numbers of cleaved caspase-3 cells in HepG2 xenografts. (D) Numbers of Ki-67 positive cells in HepG2 xenografts.

To test whether the combination of AZD7762 and doxorubicin can effectively inhibit HCC tumor growth in vivo, we treated mice bearing HCC xenografts with AZD 7762, doxorubicin, or a combination of both. Whereas either doxorubicin or AZD7762 inhibited growth of HepG2 or Huh7 xenografts, the strongest inhibition effect was observed in the combination groups (Figure 5A). We observed increased cleaved caspase-3, a marker of apoptosis, in HepG2 xenografts upon either doxorubicin or AZD7762 treatment, and the combination further increased apoptosis in the xenografts (Figure 5B and 5C). The proliferation of cancer cells in the xenografts, as indicated by the Ki-67 positive cells, decreased upon doxorubicin or AZD7762 treatment, and the combination decreased it further (Figure 5D).

## Discussion

Chronic HBV and HCV infection is believed to contribute to 80 % of HCC^23^. Several studies have reported that HBV and HCV could augment DNA damage in liver cells. The HBV X gene product HBx interferes with DNA repair by binding to damaged DNA and it sensitizes liver cells to low dose UV irradiation^24^. HBx also inhibits nucleotide excision repair through both p53-dependent and -independent mechanisms^25^,^26^. Hepatitis C virus interferes the DNA damage repair by inhibiting p53 activation via the protein phosphatase 2A catalytic subunit (PP2Ac)^27^ and by sequestering p53 in the cytoplasm through the NS2 protein^28^. On the other hand, high phosphorylated ATM, ATR, H2AX, and TP53 were found in occult HBV infection and HCV infection^29^,^30^. Elevated levels of DNA damage were also reported in hepatocellular carcinoma^31^,^32^,^33^,^34^. Virus related increase DNA damage and impairment of DNA damage repair both contribute to genomic instability during hepatocellular carcinogenesis, and in turn, induces the up-regulation of DNA repair genes. Here we show that *ATR* and *CHK1* genes are up-regulated in HBV as well as HCV positive HCCs (Figure 1), and this finding is consistent with previous reports^35^,^36^. In addition, we show the up-regulation of the *ERH* gene in HCCs, which has not been reported in HCC previously. We also show that ERH knockdown in HCC cells leads to dysregulated DNA damage response to UV irradiation. The expression of ATR is decreased in ERH knocked-down HCC cells due to reduced ATR mRNA splicing. Our findings thus imply ERH as a novel regulator of DNA damage response genes and are important for the splicing, and thus expression, of the *ATR* gene.

We observed a modest but significant increase of the proportion of comet in ERH knocked-down HepG2 cells, indicating cells with DNA damage (Figure 2C). As we have previously shown that ERH depletion resulted in mitotic defects through down-regulation of CENP-E, the DNA damage induced by ERH knocking-down may be a consequence of CENP-E dysfunction. Alternatively, this could also reflect the loss of ATR expression.

We previously demonstrated that ERH regulates mRNA splicing of CENP-E mRNA through interacting with the splicesome protein SNRPD3^11^. Here we showed that down-regulation of ERH by siRNAs also decreased the splicing efficiency of ATR mRNA that led to the loss of ATR protein. Our findings thus suggest that the in addition to CENP-E, ERH also controls the splicing of other mRNAs, and further investigation is necessary to elucidate the mechanism by which ERH operates in the cellular mRNA splicing pathway.

As no systemic chemotherapy can effectively prolong the survival of HCC patients^4^, we investigated whether target therapies can potentiate the effect of chemotherapies. The efficacy of anticancer reagents is dependent on the cellular DNA repair capacity. We hypothesized that up-regulation of DNA damage response genes such as *ERH*, *ATR* and *CHK1* in HCC tumor cells could contribute toward resistance to DNA-damaging chemotherapy and inhibiting DNA damage response might thus overcome this resistance. We used AZD 7762 to target Chk1, the major downstream effector of the ERH-ATR axis. Chk1 inhibition has been previously shown to sensitize cancer cells toward genotoxic agents in vitro and in vivo^20^,^35^,^37^,^38^,^39^,^40^. In this study, we showed that AZD7762 induces S phase arrest and sensitizes HCC cells to doxorubicin, a well-studied chemotherapy for treating HCC^4^, in vitro (Figure 4). We also observed strong inhibition of growth of HCC xenografts in mice treated with a combination of doxorubicin plus AZD7762 (Figure 5), and we observed a significant more caspase-3 positive cells, suggestive of apoptosis, in tumors from mice treated with the combination. Although the clinical development of AZD7762 was terminated due to cardiac toxicity^41^, several other Chk1 inhibitors are under development pre-clinically or clinically^42^. Our findings thus support further testing of Chk1 inhibitors in combination with doxorubicin for the treat advanced HCCs.

## Methods

### HCC cells and reagents

HepG2 and Huh7 cells were maintained in DMEM (Gibco-BRL, Gaithersburg, MD) supplemented with 10% heat inactivated fetal bovine serum (FBS), 2 mM glutamine, 100 U/ml penicillin, and 100 μg/ml streptomycin.

To generate ERH stably expressing HepG2 cells for the rescue experiment with siERH 5, the C-terminal HA-Flag–tagged human ERH cDNA was cloned into pHAGE lentiviral vector with hygromycin resistance marker. Vector plasmids were cotransfected with packaging plasmids in 293T cells, and HepG2 cells were infected with viral supernatants containing 4 μg/mL polybrene.

AZD7762 was purchased from Selleck Chemicals (Houston, TX), and doxorubicin was from Sigma-Aldrich (St. Louis MI). Compounds were solved in DMSO at concentration of 10 mM and were stored at −20°C.

### Gene expression dataset from the Gene Expression Omnibus (GEO) database analysis

The hepatocellular carcinoma patient gene expression data was available on Gene Expression Omnibus (GEO) with accession number GSE14520 and GSE6764. Expression data were normalized using the RMA method and analyzed using GeneSpring GX software (Agilent Technologies). Use and analysis of the GEO datasets were conducted in accordance with the GEO regulations.

### siRNA transfection

siERH-3 and siERH-5 were obtained from Dharmacon (Lafayette, CO) and Qiagen (Venlo, Netherlands), respectively. Control siRNA was from Qiagen. Sequence for the siRNAs were as followed: siERH #3 GAACTTATGCTGACTACGA and siERH #5 GAGGATCTTGTTCAATCGGAA. Transfection of siRNA into HCC cells was performed using Lipofetamine RNAiMAX (Invitrogen, Carlsbad, CA) following manufacturer's instruction at final concentration of 10 nM.

### Western Blot

Western blots were performed as described previously^11^. Antibodies were obtained from Abcam (ERH), Cell Signaling Technology (β-tubulin, ATR, pATR, pCHK1, r-H2Ax, PARP) and Santa Cruz (CHK1). Images of blots were captured using the FluoChem HD2 Imaging System (Alpha Innotech).

### RNA extraction and real-time polymerase chain reaction (qPCR)

Total RNA from cell lines was extracted with RNeasy Mini kit (Qiagen). The RNA was reverse transcribed to DNA using High Capacity cDNA Reverse Transcription kit (Applied Biosystems, Foster City, CA). Expression of ERH and ATR mRNA was evaluated by using Power SYBR Green PCR Master Mix (Applied Biosystems). GAPDH mRNA was used as an endogenous control. Expression of RNA was analyzed using the 2^−ΔΔCt^ method. Each RT-qPCR assay was performed in triplicates.

Primer for mRNA expression and splicing experiments were demonstrated in supplementary figure S4.

### Comet Assay

The cells were stained by using the OxiSelect™ Comet Assay Kit (Cell Biolabs, Inc. San Diego, CA) according to the manufacturer's instruction. Briefly, after exposure to UV, the cells were incubated for 0, 1 and 24 hours in DMEM. Then the cells were trypsinzed by 0.25% trypsin/EDTA and resuspended to 1 × 10^5^ cells/mL in ice-cold PBS. Combine cell samples with Comet Agarose (step 2) at 1:10 ratio, titrate to mix and immediately pipette 75 μL/well onto the OxiSelect™ Comet Slide. Slides were stored in the dark at 4°C for 15 min before adding pre-chilled lysis buffer for 45 min. The slides were immersed in freshly prepared alkaline solution (0.3 M NaOH containing 1 mM EDTA, pH >13) for 30 minutes at 4°C. Gel electrophoresis was performed at 1 V/cm for 30 minutes. The Comet slides were washed with 70% ethanol for 5 minutes, air-dried for 2.5 hours, stained with diluted Vista Green DNA Dye for 15 minutes, and then read by fluorescence microscopy using a FITC filter. At least 100 cells from each slide were analyzed. Cells were scored on a 0 to 4 scales^43^,^44^. In this study, score 1–4 cells were all calculated as comets (+) cells.

### Growth inhibition assay

HepG2 and Huh7 cells were treated with various concentrations up to 2.7 μM of AZD7762, doxorubicin, or combination for 72 hours. Cell viability was determined by the MTT [3-(4,5-dimethylthiazol-2-yl)-2,5- diphenyltetrazoliumbromide] assay (Sigma-Aldrich Co). The IC_50_ to the drug was determined using GraphPad Prism version 5 (GraphPad Software, Inc. La Jolla, CA).

To determine the synergistic effect of AZD7762 and doxorubicin, HepG2 or Huh7 cells were treated with AZD7762 and doxorubicin at 1:1 ratio. The combination index (CI) value was determined using CompuSyn software 1.0 (ComboSyn Inc. Paramus, NJ). Synergy was defined as a CI value less than 1.0^45^.

### Cell cycle analysis

Flow cytometry was used to study cell-cycle distribution. Cells were treated with AZD7762, doxorubicin or combination treatment for 72 hours. Cell cycle analyses were performed using BD FACSCalibur™ as described previously^11^.

The occurrence of apoptosis was determined by the fluorescein isothiocyanate (FITC) Annexin V Apoptosis Detection Kit (Becton, Dickinson and Company) after cells were treated with AZD7762, doxorubicin or combination treatment for 48 hours.

### Xenograft studies

Six-week-old male BALB/c nude mice were inoculated subcutaneously at flank with 5 × 10^6^ HepG2 cells or 1 × 10^7^ Huh7 cells in 100 μl PBS. When the diameter of tumors reached 5 mm, mice were randomly assigned to four treatment groups of five mice each: control (water for injection intraperitoneal (ip)), doxorubiciin (4 mg/kg twice per week ip^46^), AZD7762 (20 mg/kg/day on day1, 2, 4, and 5 per week, ip^47^), and AZD7762 + doxorubicin. The mice were treated for 2–3 weeks and the body weight and tumor volume of each mouse were monitored. Tumor volume was calculated as V = d^2^ × D/2, where d is the shortest and D is the longest diameter, respectively. Use of animals was conducted in accordance with the Institutional Animal Care and Use Committee (IACUC) guidelines of the College of Medicine, National Taiwan University and was approved by the IACUC of the College of Medicine, National Taiwan University.

### Immunohistochemistry

Frozen sections (8 μm thick) were stained by using the NoVo Link Polymer Detection System (Leica, Biosystems Newcastle Ltd, UK), followed by AEC substrate kit (Vector Laboratories Inc. Burlingame, CA), according to the manufacturer's instruction. Hematoxylin and eosin (H&E) stain and immunohistochemistry study were performed as described previously^48^. Isotype antibody was used as the staining negative control. Anti-Ki-67 and anti-caspase 3 antibodies were from Santa Cruz and Cell Signaling.

### Statistical analysis

We used the Spearman method to analyze correlations between variables. Comparisons of variables between two groups were performed using two tailed Student's t test. Paired t-test were used for comparing tumor and non-tumor part mRNA expression. For each cohort analyzed, ERH and ATR expression among three or more groups were performed using one-way ANOVA, followed by Bonferroni test for post hoc analysis. *p*-values less than 0.05 were regarded as significant.

## Author Contributions

Conception and design: M.T.W., J.L. and J.C.S. Acquisition of data: M.T.W., T.H.T., S.C.W., Y.J.H. and H.L.L. Analysis and interpretation of the data: M.T.W., J.H.L., J.M.W. and J.C.S. Writing the manuscript: M.T.W., J.H.L., J.L. and J.C.S. All authors approved the final version of the manuscript.

## Supplementary Material

Supplementary Information supplementary figures
