# Supplementary material for: Enhancer of rudimentary homolog regulates DNA damage response in hepatocellular carcinoma
Source: Sci Rep. 2015 Apr 9;5:9357. doi: 10.1038/srep09357 (PMC4399501; doi:10.1038/srep09357)

**Enhancer of rudimentary homolog regulates DNA damage response in hepatocellular carcinoma**

Meng-Tzu Weng^1,2^, Tzu-Hsun Tung^3^, Jih-Hsiang Lee^4^, Shu-Chen Wei^5^, Hang-Li Lin^5^, Yu-Jung Huang^3^ , Jau-Min Wong^5^, Ji Luo^6^, Jin-Chuan Sheu^5*^

^1^Graduate Institute of Clinical Medicine, National Taiwan University, Taipei 100, Taiwan;

^2^Far-Eastern Memorial Hospital, New Taipei 220, Taiwan,

^3^Liver Disease Prevention and Treatment Research Foundation, Taipei 100, Taiwan

^4^Clinical Trial Center, ^5^Department of Internal Medicine, National Taiwan University Hospital and College of Medicine, Taipei 100, Taiwan

^6^Cancer Systems Biology Section, Laboratory of Cancer Biology and Genetics, National Cancer Institute, NIH Bethesda, MD 20892, USA

Corresponding author:

Jin-Chuan Sheu MD PhD

Department of Internal Medicine, National Taiwan University Hospital and College of Medicine

7 Chung-Shan South Road, Taipei, Taiwan, ROC

Tel: +886-2-23123456 ext 67266

Fax: +886-2-23819723

e-mail: jcsheu@ntu.edu.tw

**Supplementary figure legends**

**Figure S1. (A) Knocking down ERH leads to increase accumulation of G2/M in HepG2 and Huh7 cells. Cells were fixed 3 days post-siRNA transfection.**

**Figure S2. (A) Knocking down ERH causes ATR mRNA expression level decreased in HCT116 cells and HCC827 cells. (B) Expression of Chk1 mRNA in HepG2 cells upon 3 days of ERH siRNA transfection. (n.s: non-specific). (C) ATR mRNA expression decreased upon 2 days of SNRPD3 siRNA transfection in Huh7 cells. (D) ATR protein level partially decreased upon 3 days of SNRPD3 siRNA transfection.**

**Figure S3. (A) Cell Viability of HCC cells to doxorubicin upon 3 days of control siRNA or ERH siRNA transfection. Cell Viability of (B) HepG2 and (C) Huh7 cells under different concentration of AZD7762, doxorubicin and combination treatment.**

**Figure S4. Sequences of primers used for (A) mRNA expression and (B) ATR mRNA splicing**

**Figure S1**


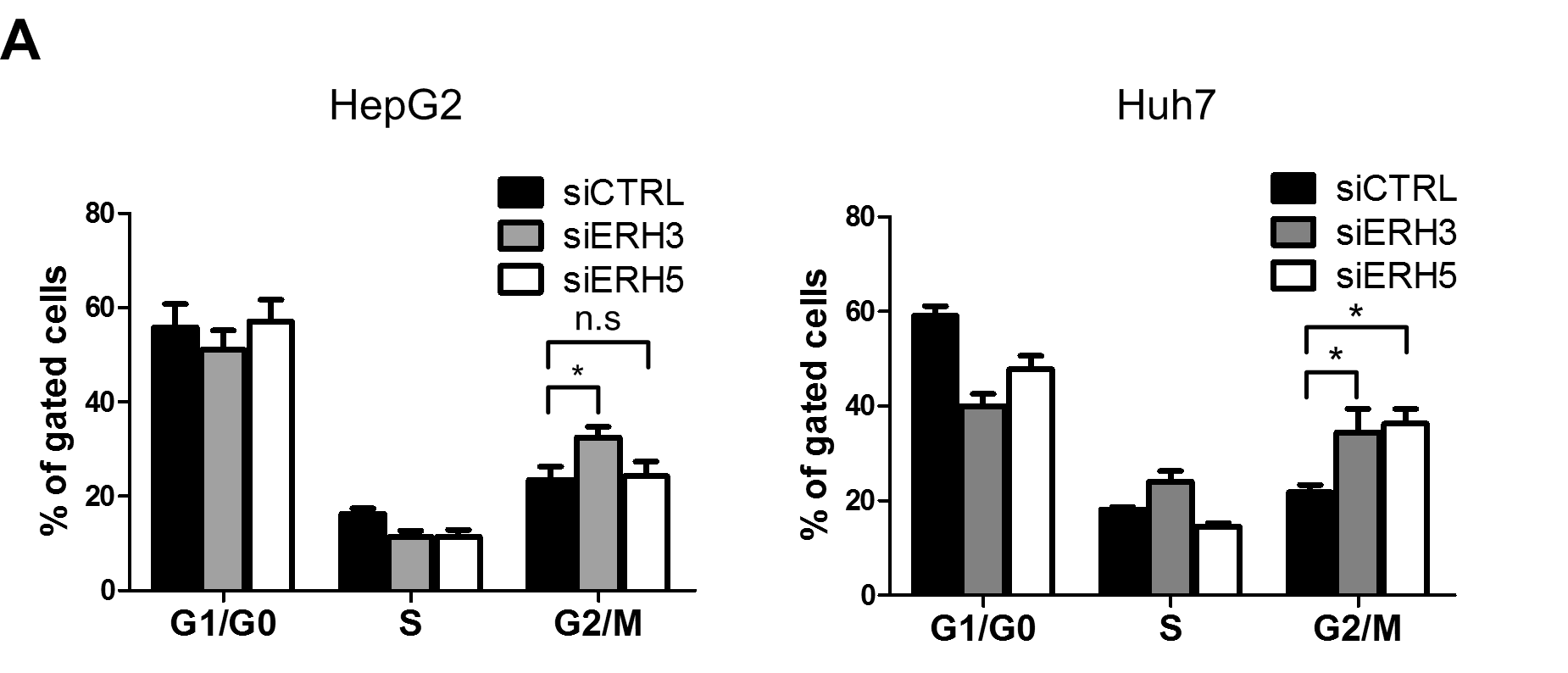


**Figure S2**


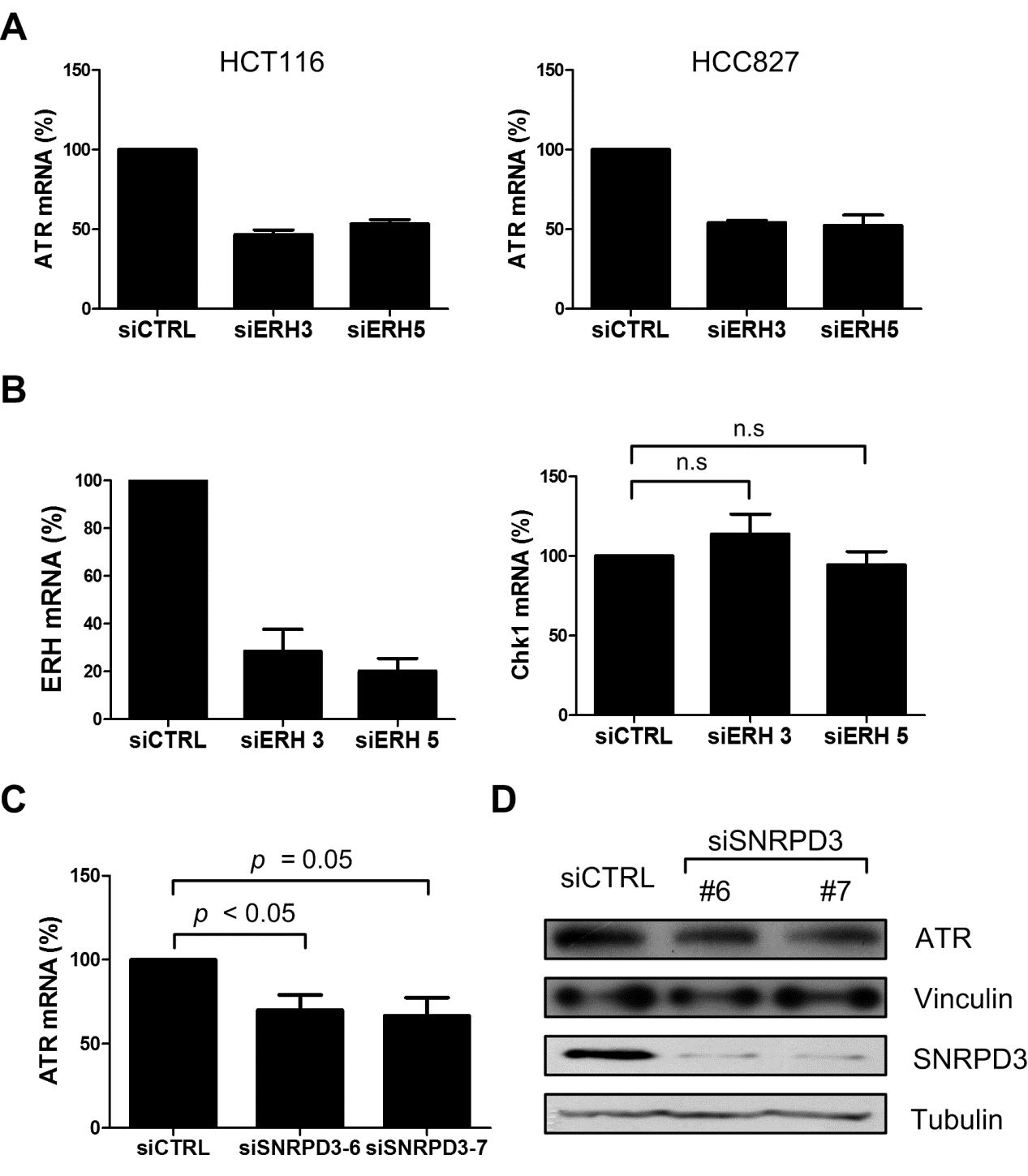


**Figure S3**


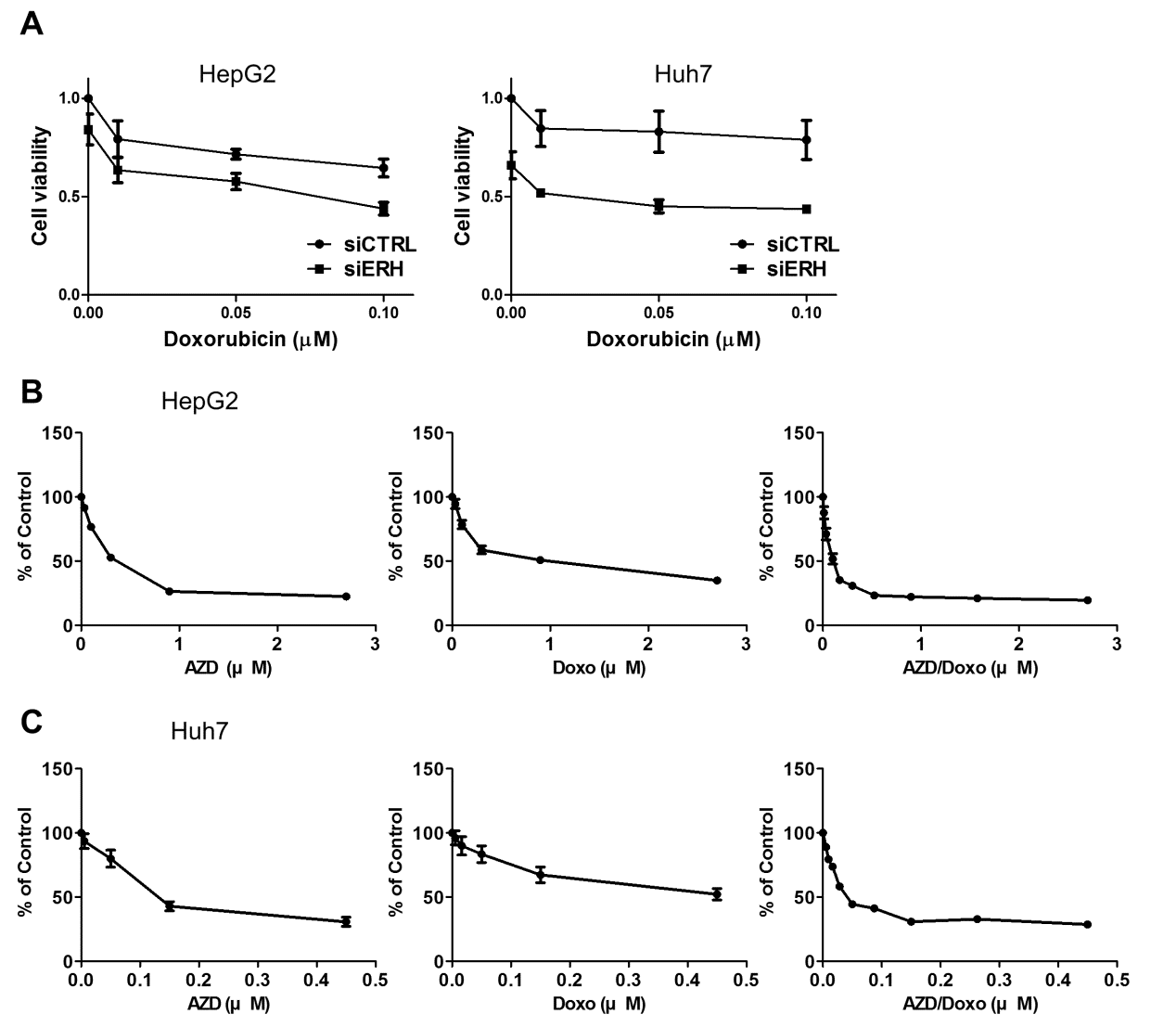


**Figure S4**


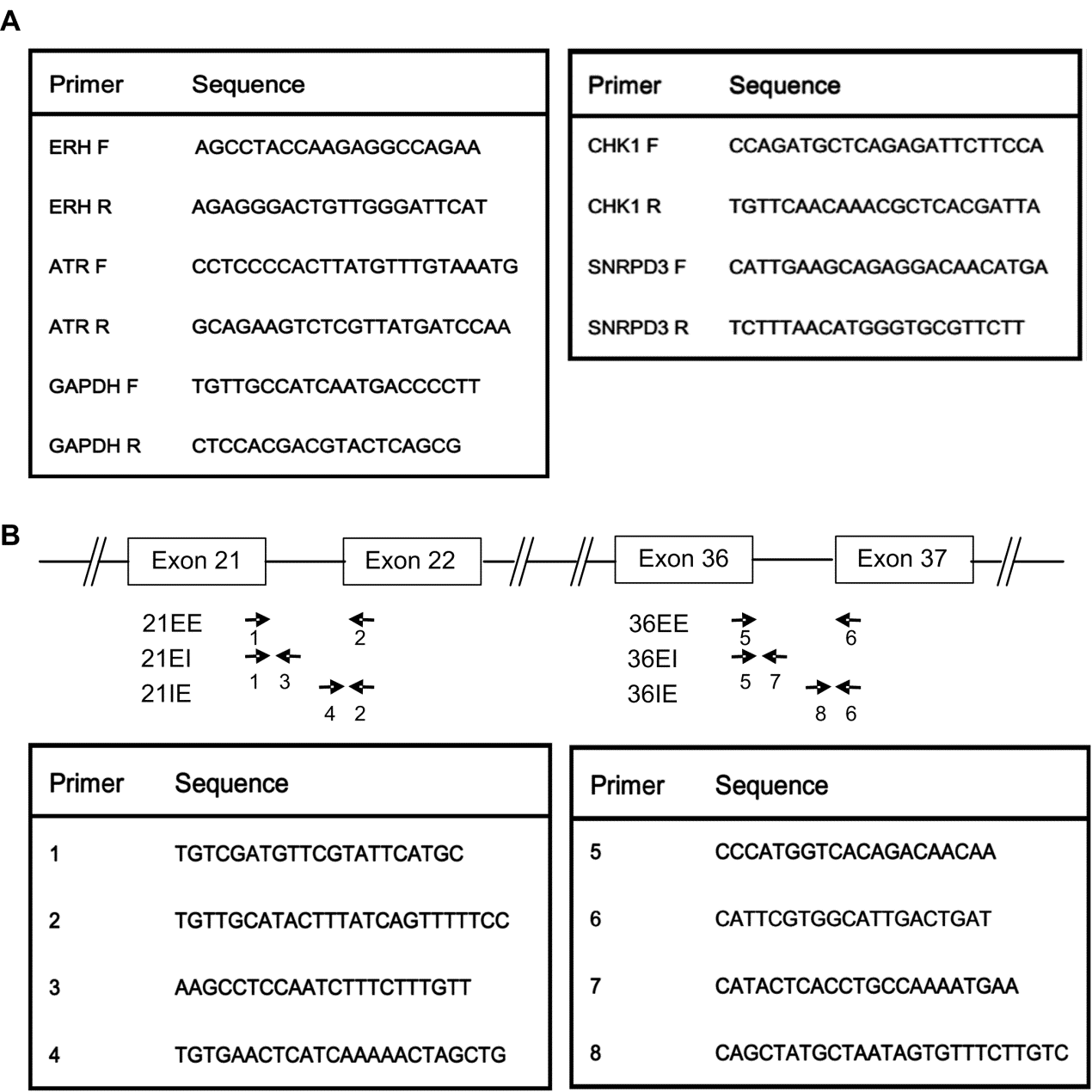

Supplement: Supplementary Information — supplementary figures [file srep09357-s1.doc]
